# Propiconazole Is a Specific and Accessible Brassinosteroid (BR) Biosynthesis Inhibitor for *Arabidopsis* and Maize

**DOI:** 10.1371/journal.pone.0036625

**Published:** 2012-05-09

**Authors:** Thomas Hartwig, Claudia Corvalan, Norman B. Best, Joshua S. Budka, Jia-Ying Zhu, Sunghwa Choe, Burkhard Schulz

**Affiliations:** 1 Department of Horticulture and Landscape Architecture, Purdue University, West Lafayette, Indiana, United States of America; 2 School of Biological Sciences, College of Natural Sciences, Seoul National University, Seoul, Korea; 3 Department of Plant Biology, Carnegie Institution for Science, Stanford, California, United States of America; 4 Plant Genomics and Breeding Institute, Seoul National University, Seoul, Korea; Umeå Plant Science Centre, Sweden

## Abstract

Brassinosteroids (BRs) are steroidal hormones that play pivotal roles during plant development. In addition to the characterization of BR deficient mutants, specific BR biosynthesis inhibitors played an essential role in the elucidation of BR function in plants. However, high costs and limited availability of common BR biosynthetic inhibitors constrain their key advantage as a species-independent tool to investigate BR function. We studied propiconazole (Pcz) as an alternative to the BR inhibitor brassinazole (Brz). *Arabidopsis* seedlings treated with Pcz phenocopied BR biosynthetic mutants. The steady state mRNA levels of BR, but not gibberellic acid (GA), regulated genes increased proportional to the concentrations of Pcz. Moreover, root inhibition and Pcz-induced expression of BR biosynthetic genes were rescued by 24epi-brassinolide, but not by GA_3_ co-applications. Maize seedlings treated with Pcz showed impaired mesocotyl, coleoptile, and true leaf elongation. Interestingly, the genetic background strongly impacted the tissue specific sensitivity towards Pcz. Based on these findings we conclude that Pcz is a potent and specific inhibitor of BR biosynthesis and an alternative to Brz. The reduced cost and increased availability of Pcz, compared to Brz, opens new possibilities to study BR function in larger crop species.

## Introduction

Brassinosteroids (BRs) are poly-hydroxylated steroidal hormones with profound effects on several physiological plant responses. They are involved in regulating cell elongation and division [1]–[2], vascular differentiation [3]–[4], photomorphogenesis [5]–[6], leaf angle inclination [7]–[8], seed germination [9]–[10], stomata development [11], as well as suppression of leaf senescence and abscission [12]. Radioactive tracer studies in cell cultures of *Catharanthus roseus* established the steps of the BR metabolic pathway [13]. This work was complemented by the characterization of several BR-deficient mutants in *Arabidopsis* [14]–[20], as well as crops like tomato, pea, and rice [21]. These studies showed that several steps of BR biosynthesis are mediated by cytochrome P450 monooxygenases (P450s) [13], [21]. Although the importance of BRs for agricultural crops such as sorghum (*Sorghum bicolor*) and maize (*Zea mays* L.) has been recognized [22], only a few null-mutations have been reported in these species [23]–[24].

The field of chemical genomics greatly benefited from the use of chemical inhibitors/modifiers [25]–[26]. Potent and specific biosynthesis inhibitors are useful tools to evaluate the functions of endogenous substances, including phytohormones. Biosynthetic mutants and specific metabolic inhibitors displayed their effectiveness in mode of action studies of gibberellic acid (GA) and BRs [27]–[28].

**Figure 1:**
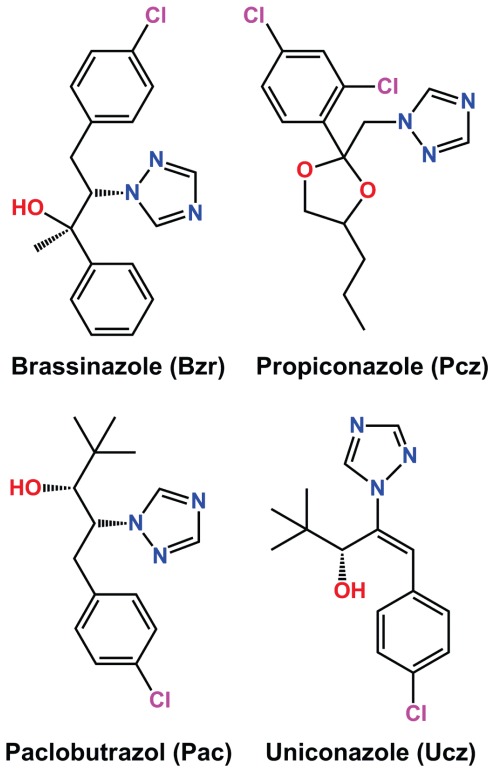
Chemical structures of brassinazole, propiconazole, paclobutrazole, and uniconazole. Structure elements critical for inhibitor activity have been color-coded: (blue) nitrogen atoms in the azole ring; (purple) chlorine atom(s) of the phenyl ring; and (red) either primary/secondary hydroxyl group or 1,3-dioxlane. Structures were drawn using the ChemBioDraw 12.0.2 software and structures were compared to the ChemACX 12.12.1 database.

Numerous triazole compounds have been shown to inhibit P450s, one of the largest and most ubiquitous group of plant enzymes that catalyze oxidative processes in life systems [29]. Paclobutrazol (Pac) and uniconazole (Ucz) are two triazole plant growth regulators (Fig. 1) that block sterol 14R-demethylation, phenocopy GA mutants, and reduce endogenous GA levels [30]. Both compounds inhibit P450 CYP701, which catalyzes an early step in GA biosynthesis [30]. Furthermore, Ucz also has been reported to slightly decrease the endogenous concentration of castasterone and inhibits BR-induced tracheary element differentiation [31]–[32]. These reports suggested that Ucz may also affects BR biosynthesis and later screens of structurally similar azoles led to the development of brassinazole (Brz) (Fig. 1), the first specific BR biosynthetic inhibitor [33]–[34].

Brz's mode of action is to bind and block DWF4/CYP90B1 [35]. DWF4 is a P450 that mediates multiple rate-limiting C22 alpha-hydroxylations in the biosynthesis of BRs [35]. *DWF4* expression is a target of regulation by both endogenous signals like auxin [36]–[37] and exogenous cues like temperature [38]. Brz and its even more specific derivative Brz2001 [39] became invaluable tools for BR research. Not only did they help to reveal the role of BRs in various plant species [40], they were also essential for the isolation and characterization of genes that function in BR signaling [41]–[43]. However, low accessibility and high costs associated with Brz/Brz2001 have limited their use in agricultural crops that often require large-scale applications. In this context, it would be beneficial to the research community to have access to potent, specific, and more cost efficient azole BR inhibitors in plants.

The triazole compound propiconazole (Pcz), 1-[ [2-(2,4-dichlorophenyl)-4-propyl-1,3-dioxolan-2-yl]methyl]-1,2,4-triazole, (Fig. 1) as a potent inhibitor of BR biosynthesis was first reported after examining its inhibitory effect on hypocotyl elongation of cress plants (*Lepidium sativum*) [27]. This inhibitory effect of Pcz was reversed by co-application with brassinolide. Based on the Pcz structure additional BR inhibitors, such as 2RS,4RS-1-[2-(4-trifluoromethylphenyl)-4-n-propyl-1,3-dioxolan-2-ylmethyl]-1H-1,2,4-triazole, were identified [27]. On the other hand, Pcz has been commercially used as fungistat (BannerMaxx, Syngenta) against a broad range of phytopathogenic fungi. Its fungistatic mode of action is the same as that of Ucz and Pac; blocking of lanosterol 14R-demethylase (CYP51A1) [44]–[45]. Pcz has also been studied extensively for its toxicity on plants, animals, humans, and the environment [46]–[47]. Here we present a molecular genetic analysis of Pcz's effects on *Arabidopsis* and maize seedlings. Our results indicate that Pcz is a potent and specific inhibitor of the BR metabolic pathway in plants.

## Results

### *Arabidopsis* seedlings treated with Pcz display dwarf phenotypes

**Figure 2:**
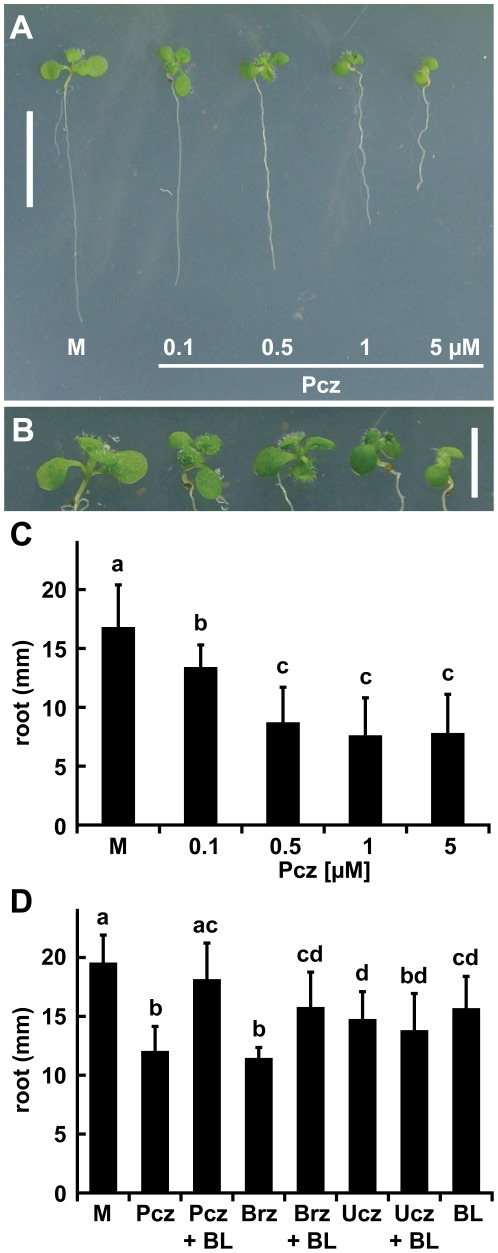
Responses of *Arabidopsis* seedlings to inhibitor treatments and BL Complementation. (**A–C**) 3-day old Ws-2 seedlings were transferred to ½ MS media containing 0, 0.1, 0.5, 1, or 5 µM of Pcz and incubated for 5 more days. (**A**) Seedlings at the end of treatment (5 d). (**B**) Close-up of the cotyledons and true leaves in the same order as (**A**). (**C**) Average root lengths at day 3 of treatment are illustrated (*n*>15). (**D**) 3-day old Ws-2 seedlings were transferred to ½ MS media containing 1 µM of Pcz, Brz, Ucz, or co-applications of inhibitors (1 µM) with 0.1 µM BL or 0.1 µM BL alone and incubated for 4 more days. Average root lengths at the end of treatment (n>15). (**C–D**) Error bars represent standard deviation and lowercase letters indicate significant differences among treatments determined by “Post-hoc” test. Scale bar (**A**) 1 cm and (**B**) 0.5 cm.

To study Pcz's impact on *Arabidopsis*, we treated wild-type Ws-2 plants with Pcz concentrations ranging from 0.1 to 5 µM for 5 days. Cotyledons showed a reduction in size and epinatic growth responses with treatments of Pcz (Fig. 2A–B). Subsequently, we evaluated the effect of Pcz on primary root length. The results showed a dose-dependent reduction of primary root growth, where 0.1 µM Pcz decreased the elongation by 20% and 5 µM Pcz by 54% compared to mock conditions (Fig. 2A, 2C). No further significant decrease was observed at concentrations higher than 0.5 µM Pcz (Fig. 2C).

We then evaluated the overall efficacy and specificity of Pcz compared to equal concentrations of Brz and Ucz. Wild-type Ws-2 plants treated with 1 µM Pcz or Brz for 4 days showed comparable reductions in root length of 38% and 41%, respectively (Fig. 2D). In contrast, the impact of 1 µM Ucz on reduction of root length was significantly less with only a 25% decrease compared to mock treatments (Fig. 2D). We found a complementation of the phenotypes induced by 1 µM Pcz or Brz, but not 1 µM Ucz, with co-applications of 0.1 µM 24epi-brassinolide (BL), a bioactive epimer of brassinolide (Fig. 2D). Root length of plants co-treated with 1 µM Pcz plus 0.1 µM BL was not significantly different from mock treatments (Fig. 2D). On the contrary, the co-application of 1 µM Ucz and 0.1 µM BL showed no significant difference to treatments with 1 µM Ucz alone (Fig. 2D). These results suggest that Pcz and Brz have comparable efficacies in the inhibition of the BR biosynthetic pathway.

**Figure 3:**
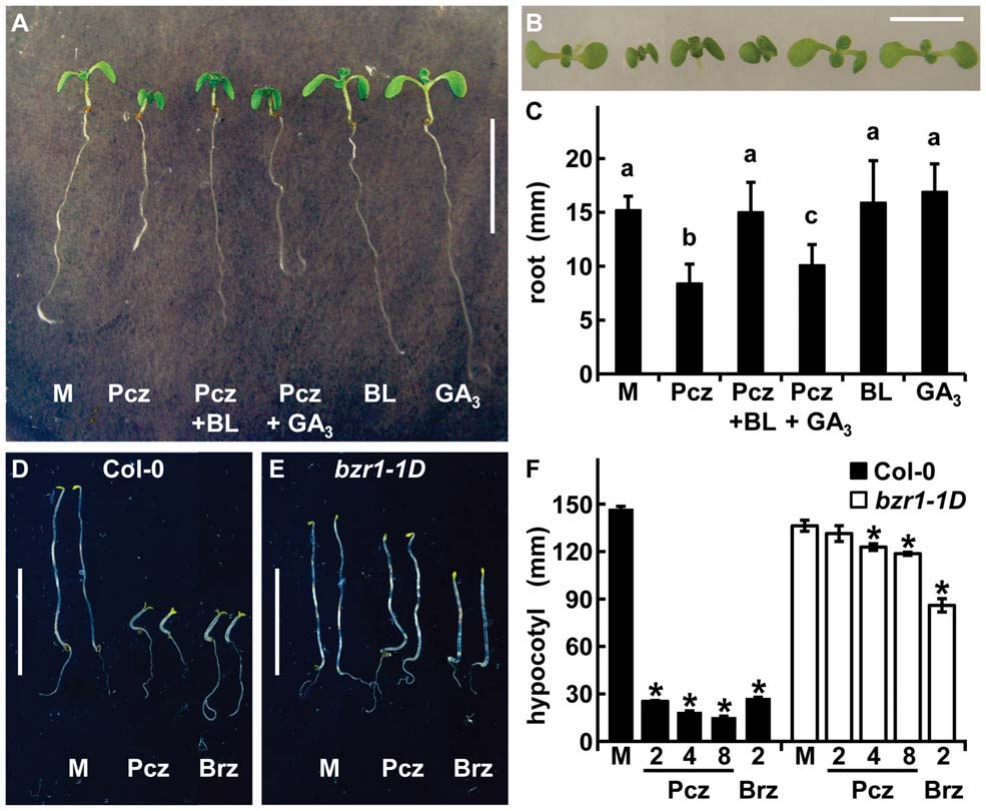
Pcz specificity towards BR biosynthesis inhibition. (**A–C**) 3-day old Ws-2 were transferred to ½ MS media containing either 1 µM Pcz, 0.1 µM BL, 10 µM GA_3_, or co-applications of 1 µM Pcz with 0.1 µM BL or 10 µM GA_3_ and grown for 3 more days. (**A**) Seedlings at the end of treatment. (**B**) Close-up of the cotyledons and true leaves in the same order as (**A**). (**C**) Average root lengths at day 3 of treatment (*n*>15). (**D–F**) Col-0 and *bzr1-1D* (Col-0) grown on ½ MS media in the dark for 7 days. (**D**) Col-0 or (**E**) *bzr1-1D* grown on medium with 2 µM Pcz or 2 µM Brz. (**F**) Hypocotyl lengths of Col-0 and *bzr1-1D* grown on medium with 2, 4, 8 µM Pcz or 2 µM Brz (n≥10). (**C**, **F**) Error bars represent standard deviation. (**C**) Lowercase letters indicate significant differences among treatments determined by “Post-hoc” test (*p*<0.05). (**F**) Asterisks indicate significant difference to the respective mock determined by Student's *t*-test (*p*<0.01). Scale bar (**A**, **D**, **E**) 1 cm and (**B**) 0.5 cm.

In some cases, triazole derivatives affect multiple targets; although usually to a varying extent [48]. To address whether Pcz inhibition is specific to the BR biosynthetic pathway or also affects P450 monooxgenases in GA biosynthesis, we compared co-applications of Pcz with BL or GA_3_. Again, *Arabidopsis* seedlings co-treated with Pcz and BL showed no difference in primary root length compared to mock conditions (Fig. 3A, 3C). In contrast, Pcz induced-inhibition of root length was not recovered by exogenous co-application of GA_3_ (Fig. 3A, 3C). We did find a slight increase (17%) in root length with the co-application of Pcz and GA_3_ relative to Pcz treatment, however, a similar increase (11%) was found for GA_3_ application compared to mock. Taken together, the results indicate that the inhibition of root growth caused by Pcz is complemented by the co-application of BL, but not GA_3_.

*Arabidopsis brassinazole resistant1-1D* (*bzr1-1D*) mutants carry a dominant gain of function mutation in the transcription factor and major component of BR signaling, *BZR1*, which results in a constitutive BR response even in the absence of BRs. Therefore, *bzr1-1D* mutants are sensitive indicators for BR inhibitor specificity [49]. Wild-type (Col-0) plants treated with 2 µM Pcz showed a reduction in hypocotyl length of 83%, whereas *bzr1-1D* mutants exhibited only 4% shorter hypocotyls, compared to mock (Fig. 3D–F). Although *bzr1-1D* plants were more resistant to Brz applications than wild type, 2 µM Brz did reduce the length of *bzr1-1D* hypocotyls by 37% compared to mock (Fig. 3E–F).

### BR biosynthesis mutants show reduced sensitivity towards Pcz

**Figure 4:**
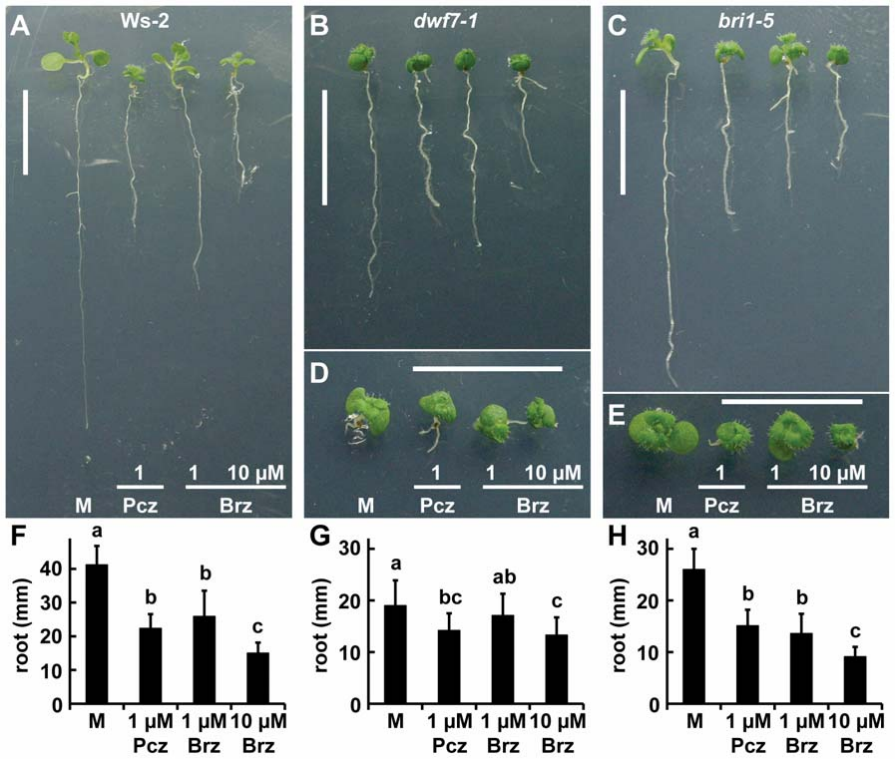
Sensitivity of *Arabidopsis* wild type, BR biosynthetic mutant *dwf7-1*, and signaling mutant *bri1-5* to Pcz and Brz. (**A–C**) Seedlings of (**A**) Ws-2, (**B**) the BR biosynthetic mutant *dwf7-1* (Ws-2), and (**C**) the BR signaling mutant *bri1-5* (Ws-2) grown on ½ MS media with either 1 µM Pcz or Brz or 10 µM Brz. (**D–E**) Close-up of the shoot apices harboring cotyledons and the first pair of true leaves of (**D**) *dwf7-1* and (**E**) *bri1-5*. (**F–H**) Average root lengths of (**F**) Ws-2, (**G**) *dwf7-1* and (**H**) *bri1-5* measured at the end of treatments (*n*>10). (**F–H**) Error bars represent standard deviation and lowercase letters indicate significant differences among treatments determined by “Post-hoc” test (*p*<0.05). Scale bar (**A–E**) 1 cm.

The azole ring of Brz binds to the heme prosthetic group of DWF4 (CYP90B1), the rate-limiting enzyme in BR biosynthesis, forming a coordination complex, which impairs DWF4 activity [35]. Mutants already deficient in BR biosynthesis should thus show a reduced sensitivity towards BR biosynthetic inhibitors such as Brz and Pcz. We tested wild-type and *dwf7-1* seedlings with Pcz and compared their responses to Brz treatments (Fig. 4). DWF7 catalyzes the conversion of episterol to 5-hydroepisterol upstream of DWF4 [18]. Wild-type (Ws-2) plants treated with either 1 µM Pcz or Brz, or 10 µM Brz produced 46%, 37%, and 64% shorter roots compared to mock conditions, respectively (Fig. 4A, 4F). Although *dwf7-1* showed a significant response to 1 µM Pcz and 10 µM Brz treatments, the relative reduction (25% and 30%, respectively) was still lower than Pcz/Brz treated Ws-2 (Fig. 4B, 4G). *bri1-5*, a weak allele of the major BR receptor *BRASSINOSTEROID RESISTENT1* (*BRI1*), and wild type respond similarly to Brz, but not to BL treatment [50]–[51]. This suggests that although *bri1-5* is affected in BR signal transduction, it still responds to changes in BR homeostasis. We found that Pcz and Brz, at equal concentrations, had similar effect on *bri1-5* and Ws-2 (Fig. 4A, 4C, 4F, 4H).

**Figure 5:**
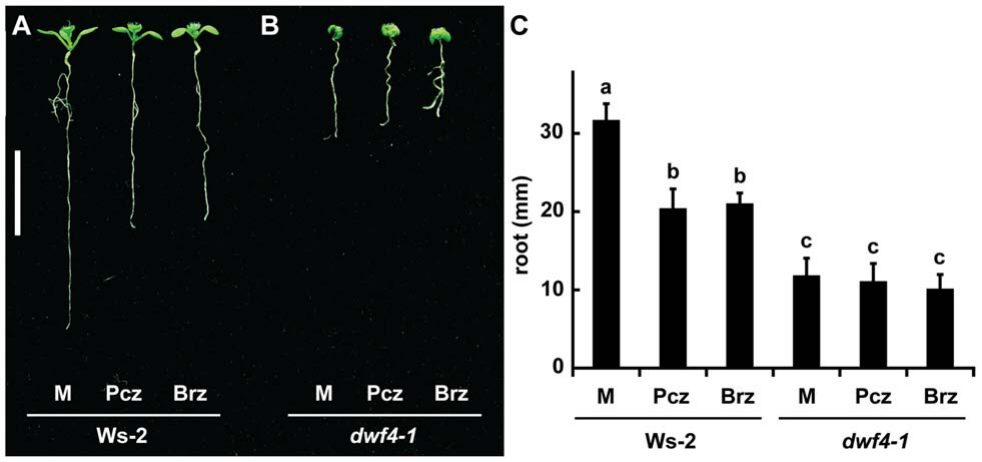
Resistance of *dwf4-1* to Pcz and Brz. (**A–B**) Seedlings of (**A**) Ws-2 and (**B**) *dwf4-1* grown on ½ MS media for 7 days, and then transferred to media containing 1 µM Pcz or Brz for 3 more days of growth. (**C**) Average root lengths of Ws-2 and *dwf4-1* measured at the end of treatments (*n*>10). Error bars represent standard deviation and lowercase letters indicate significant differences among treatments determined by “Post-hoc” test (*p*<0.05). Scale bar (**A**–**B**) 1 cm.

Since DWF4 has been shown to be a direct target of Brz [35], which is structurally similar to Pcz (Fig. 1), we also took a closer look at the effect of Pcz on *dwf4* mutants. Ws-2 plants again showed a decrease in root length upon Pcz or Brz treatment (Fig. 5A, 5C), but *dwf4-1* mutants did not exhibit a significant reduction (Fig. 5B, 5C).

### Pcz specifically influences the expression of BR biosynthetic genes

Transcriptional feedback regulation of rate-limiting step enzymes is a critical mechanism to maintain hormone homeostasis [52]–[53]. Since triazole derivatives can have multiple targets, we evaluated the expression levels of both BR (*DWF4*, *BR6ox2*, *CPD*, *BAS1* and *BZR1*) and GA (*GA20ox1* and *GA2ox1*) related genes, to further assess the specificity of Pcz.

**Figure 6:**
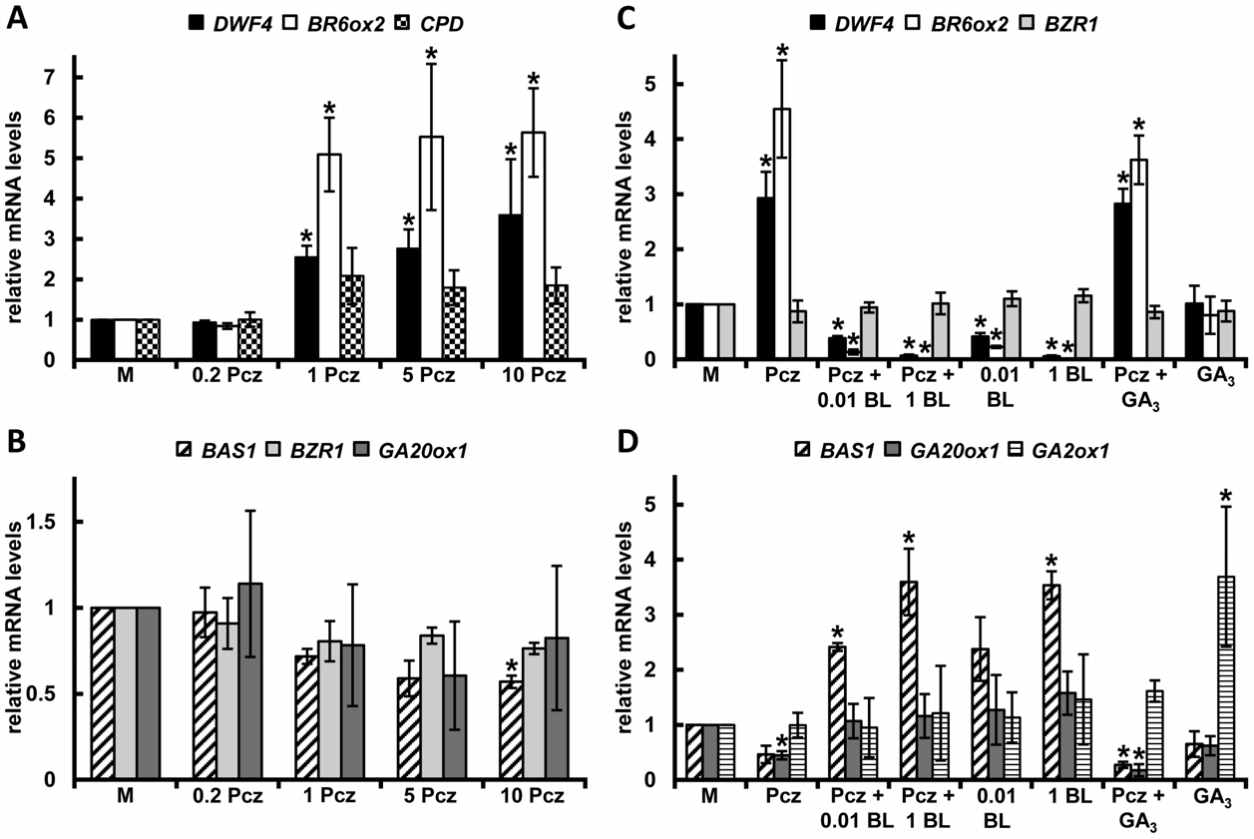
Pcz impact on the expression of BR- and GA-related genes. (**A–D**) Quantitative real-time PCR analysis of the transcript levels after treatment with 0.2, 1, 5 or 10 µM Pcz of (**A**) BR biosynthetic genes *DWF4*, *BR6ox2*, and *CPD* or (**B**) *BAS1*, *BZR1*, and *GA20ox1*, involved in BR degradation, BR signaling and GA biosynthesis, respectively. (**C**, **D**) Expression pattern of (**C**) BR biosynthesis and signaling or (**D**) BR degradation, GA biosynthesis and GA degradation (*GA2ox1*) genes measured after treatments with 1.5 µM Pcz, 0.01 or 1 µM BL, or 1 µM GA_3_ or co-applications of Pcz (1.5 µM) with 0.01 or 1 µM BL or 1 µM GA_3_. (**A–D**) Data points represent the average of three independent biological replicates with three technical replicates each. Error bars represent standard deviation. Asterisks indicate significant differences to the respective mock determined by Student's *t*-test (*p*<0.05). Ubiquitin conjugating enzyme 21 (*UBC21*) was used as internal control.

Using quantitative real-time PCR (qRT-PCR) we found a dose-dependent induction of BR biosynthetic gene expression (*DWF4*, *BR6ox2*, and *CPD*) with increasing Pcz concentrations (Fig. 6A). With the same treatment conditions the mRNA levels of *BAS1*, a gene involved in BR degradation [5], showed a downward trend (Fig. 6B). BZR1, a key regulator of BR signaling, did not show relevant changes in its expression upon Pcz treatments (Fig. 6B). The GA biosynthesis gene *GA20ox1*, which is feedback regulated by endogenous GA levels [30], also showed no increase in expression upon Pcz application (Fig. 6B).

The Pcz-induced increase in mRNA accumulation of *DWF4* and *BR6ox2* and concomitant decrease in *BAS1* expression was offset by co-application of Pcz with BL, but not by Pcz with GA_3_ (Fig. 6C). Our results also showed an expected decrease in *DWF4* and *BR6ox2* expression upon BL and to a smaller extent *GA20ox1* upon GA_3_ treatment (*p* = 0.06) (Fig. 6C–D). Together these findings suggest that Pcz specifically affects BR regulated genes and do not provide evidence for an inhibitory effect on GA biosynthesis.

### Pcz induces dwarfism in dark and light grown maize inbred W22 seedlings

**Figure 7:**
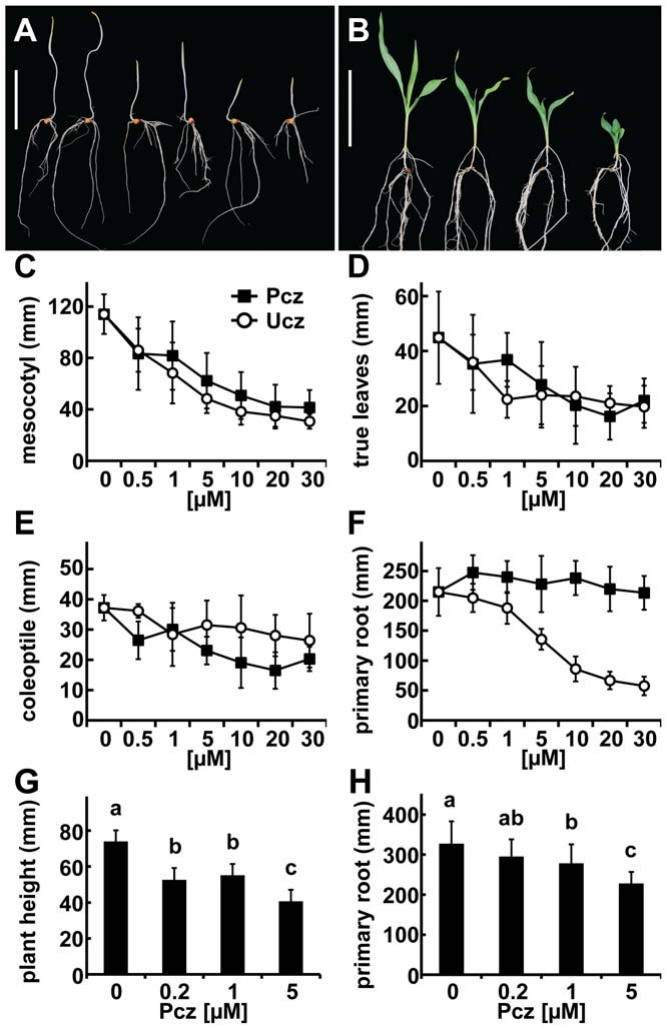
Tissue-specific response of dark and light grown W22 seedlings to Pcz and Ucz. (**A**) Maize seedlings grown for 8 d at 29°C in the dark treated with (left to right): 0 µM Pcz, 0 µM Ucz, 1 µM Pcz, 1 µM Ucz, 10 µM Pcz, and 10 µM Ucz, respectively. (**B**) W22 seedlings grown in the light for 3 weeks at concentrations of (left to right): 0, 0.2, 1, and 5 µM Pcz, respectively. (**C–F**) W22 seedlings grown for 8 d at 29°C in the dark with Pcz or Ucz at concentrations of 0, 0.5, 1, 5, 10, 20, or 30 µM. Lengths of the (**C**) mesocotyl, (**D**) true leaves, (**E**) coleoptile, and (**F**) primary root of W22 seedlings grown in the dark with indicated concentrations of Pcz or Ucz (*n*>15). (**G–H**) Analysis of W22 maize seedlings grown in the light for 3 weeks at concentrations of 0, 0.2, 1, or 5 µM Pcz (Table S5). (**G**) Plant height and (**H**) primary root length was measured (*n*>15). Error bars represent standard deviation. (**C–H**) Statistical analysis determined by “Post-hoc” test is shown (**C–F**) in Table S1 and (**G–H**) indicated by lowercase letters (*p*<0.05). Scale bar (**A–B**) 10 cm.

Studies on crop species such as sorghum, rice, and maize often require large amounts of growth media which limits the use of cost intensive inhibitors such as Brz. For that reason, we tested the effect of Pcz on both dark and light grown seedlings of the maize inbred line W22. A strong reduction of hypocotyl elongation is one of the most striking characteristics of the de-etiolation phenotype of dark grown *Arabidopsis* mutants deficient in BR biosynthesis [53]. Compared to mock, W22 seedlings grown in the dark for 8 d in the presence of 0.5 to 30 µM Pcz showed decreased mesocotyl elongation ranging from 27% to 64%, respectively (Fig. 7A, 7C). Ucz treatment of 0.5 to 30 µM reduced the length of the mesocotyl 25% to 73% (Fig. 7C). Similar to the mesocotyl, the lengths of true leaves were reduced from 21% to 51% by Pcz and from 20% to 56% by Ucz, respectively (Fig. 7D). Although both Pcz and Ucz treatment affected the coleoptile, the relative reduction in length was less pronounced than in mesocotyls and true leaves (Fig. 7E). Surprisingly, the primary root of W22 seedlings showed significant differences in sensitivity towards Pcz and Ucz. Pcz concentrations up to 30 µM had no significant effect on primary root length. In contrast, seedlings treated with equal or greater than 5 µM Ucz resulted in significantly shorter primary roots when compared to mock (Fig. 7F), reaching a reduction of 73% at 30 µM.

Pcz treatment also induced dwarfism in light grown W22 seedlings. Plants treated with 0.2 to 5 µM Pcz decreased their overall height by 29% to 45%, respectively (Fig. 7B, 7G). In addition to the dwarf stature, Pcz induced shorter leaves and resulted in a more overall compact appearance (Fig. 7B). Interestingly, unlike in dark treatments, the primary root length of seedlings grown for 21 d in the light was reduced by 30% when treated with 5 µM Pcz relative to mock (Fig. 7H).

### The genetic background of maize influences tissue specific sensitivity to Pcz

**Figure 8:**
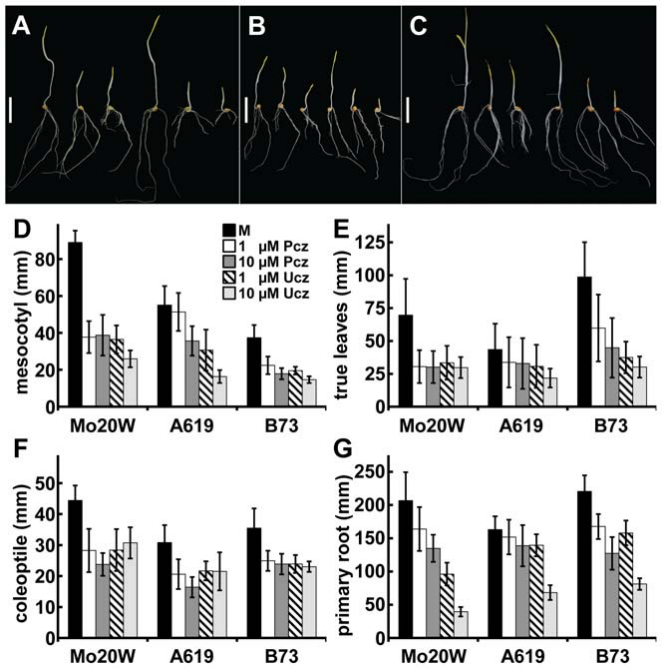
Impact of the genetic diversity on the tissue specific sensitivity towards Pcz and Ucz. (**A–C**) Maize inbred seedlings (**A**) Mo20W, (**B**) A619, and (**C**) B73 grown in vermiculite for 8 d at 29°C in the dark treated with (left to right): 0 µM Pcz, 1 µM Pcz, 10 µM Pcz, 0 µM Ucz, 1 µM Ucz, and 10 µM Ucz, respectively. (**D–G**) Length of the (**D**) mesocotyl, (**E**) true leaves, (**F**) coleoptile, and (**G**) primary root of Mo20W, A619, and B73 maize seedlings treated with 0 µM Pcz, 1 µM Pcz, 10 µM Pcz, 0 µM Ucz, 1 µM Ucz, or 10 µM Ucz, respectively (*n*>15). (**D–G**) Error bars represent standard deviation. Statistical analysis determined by “Post-hoc” test is shown in Table S2 (*p*<0.05). Scale bar (**A–C**) 6 cm.

Given the great diversity between maize inbred lines [54], we assessed whether the genetic background influences the effects of Pcz and Ucz on dark grown maize seedlings. Thus, we repeated the dark assay using 3 additional maize inbred lines: Mo20W, A619, and B73. The length of the four evaluated tissues (mesocotyl, true leaves, coleoptile, and primary root) showed significant differences between Mo20W, A619, and B73 even in the absence of Pcz or Ucz (Fig. 8A–G). In addition, when treated with Pcz or Ucz we observed significant differences between the inbred lines in both their overall and tissue-specific sensitivity. In the presence of 1 or 10 µM Pcz the mesocotyl length of Mo20W was reduced by 57% and 56%, respectively, relative to mock treatment (Fig. 8A, 8D). Likewise, B73 treated with the same concentrations of Pcz exhibited 40% and 52% shorter mesocotyls, respectively (Fig. 8C–D). However, in the case of A619, only 10 µM Pcz had a significant but smaller impact (35%) on mesocotyl elongation, relative to mock (Fig. 8B, 8D). Comparable results were obtained for the response of true leaves to Pcz and Ucz treatments (Fig. 8A–C, 8E).

The primary roots of Mo20W, A619, and B73 showed a significant reduction in length when treated with 1 or 10 µM Ucz (Fig. 8A–C, 8G). Interestingly, Mo20W and B73 showed, in addition to the Ucz sensitivity, a decrease in primary root length when treated with 1 or 10 µM Pcz (Fig. 8A, 8C, 8G). The primary root length of A619 was reduced significantly only at 10 µM Pcz, but to a smaller extent than Mo20W and B73 (Fig. 8A–C, 8G). The coleoptile was the only tissue evaluated with comparable responses to both Pcz and Ucz in all three inbred lines (Fig. 8A–C, 8F).

**Figure 9:**
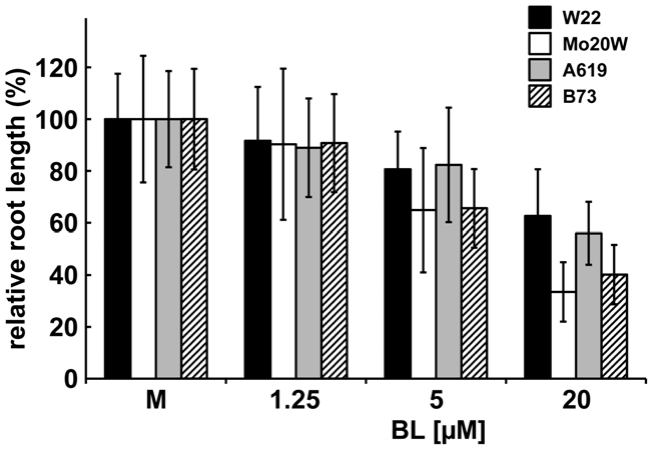
Influence of the genetic variation on the BR sensitivity in maize root. Maize inbred seedlings W22, Mo20W, A619, and B73 grown in vermiculite for 8 d at 29°C in the dark in the presence or absences of 1.25, 5, or 20 µM BL, respectively. Average length of the primary root measured at the end of treatment (*n*>15). Error bars represent standard deviation. Statistical analysis determined by “Post-hoc” test (*p*<0.05) is shown in Table S3.

To test whether the differences in Pcz efficacy are due to differences in BR response, we compared the effect of BL on root elongation between the maize inbreds. W22 and A619 plants treated with 20 µM BL exhibited the smallest relative reduction (37% and 44%, respectively) in root length (Fig. 9). On the other hand, Mo20W and B73 inbreds treated with the same amount of BL had 67% and 60% shorter roots than mock, respectively (Fig. 9). This result is consistent with our previous findings for Pcz sensitivity, and supports our hypothesis that genetic diversity influences BR responses in maize.

## Discussion

Phytohormone biosynthesis inhibitors allow the species-independent study of hormonal function during plant development. Inhibitor studies can also support the isolation and characterization of hormone deficient mutants without prior knowledge of the mutant phenotype. Pcz has previously been reported to impair the hypocotyl growth of cress seedlings and that this inhibition is reversible by the co-application of BL [27]. Chemical modification of Pcz also revealed structural elements essential for its inhibitory properties [27]. Pcz's high accessibility and economical aspects prompted us to conduct a comparative analysis with the established BR inhibitor Brz.

Pcz treatment of *Arabidopsis* seedlings produced typical BR-deficient phenotypes such as: epinastically growing and dark-green cotyledons, reduced hypocotyl length, and a significantly shorter primary root (Fig. 2). Using root length as a reference we found that even relatively low Pcz levels of 0.5 µM resulted in strong inhibition (Fig. 2). As shown in independent experiments, the impairment of root growth in *Arabidopsis* through Pcz treatment can essentially be restored to length of mock-treated seedlings by BL, but not GA_3_ (Fig. 3). The slight effect of GA_3_ on root elongation was independent of Pcz treatment and may not indicate a recovery of Pcz inhibition. In contrast, BL treatment had a dramatic effect on root elongation in Pcz treated seedlings (Fig. 3).

In the absence of BR, the transcription factor BZR1 and its homolog BZR2 (BES1) are phosphorylated by the GSK3/SHAGGY-like protein kinase BIN2 [49], [55]–[56]. Phosphorylation negates BZR1's DNA-binding capacity and increases its cytoplasmic retention by phosphopeptide-binding 14-3-3 proteins [57]–[58]. The dominant *bzr1-1D* mutation increases BZR1's dephosphorylation by the phosphatase PP2A [59]. BZR1 therefore remains nuclear localized and stabilized even in the absence of BRs causing *bzr1-1D* plants to show a constitutive BR response [49], [59]. In contrast to wild type, *bzr1-1D* mutants showed only a minor inhibition of hypocotyl growth in the presence of Pcz (Fig. 3). Current evidence in rice and cress suggests that Brz inhibits BR biosynthesis but also affects GA responses [39]. We found that *bzr1-1D* plants were more sensitive to Brz than Pcz (Fig. 3) which suggests that Pcz is more specific. This hypothesis is supported by our finding that roots co-treated with Brz and BL, but not with Pcz and BL, were significantly shorter than mock (Fig. 2).

Ucz has been extensively studied as an inhibitor of GA biosynthesis [30]. Circumstantial evidence reported by Yokota et al. [31] and Iwasaki and Shibaoka [32] indicates that Ucz might also act as a demethylase inhibitor in BR biosynthesis. We observed shorter roots and hypocotyls of Ucz-treated *Arabidopsis* seedlings. Although Pcz- and Ucz-induced phenotypes were similar, co-application of Ucz and BL was not significantly different to Ucz alone (Fig. 2). Based on these analyses, Pcz-mediated suppression of root and hypocotyl elongation is likely the result of a specific inhibition of the BR biosynthetic pathway.

DWF7/STE1 is a Δ^7^ sterol C-5 desaturase that converts avenasterol to dehydroavenasterol or episterol to 5-dehydroepisterol early in BR biosynthesis [18]. Significant reduction of root elongation with Pcz treatment was found in wild type and to lesser extent also for *dwf7-1* seedlings, whereas roots of *dwf4-1* mutants did not exhibit a significant decrease (Fig. 4, Fig. 5). The BR metabolic pathway is likely non-linear, as downstream BR intermediates can be found in most monogenic BR biosynthetic mutants, including *dwf7-1* [18], [20], [60]. Therefore, Pcz treatment may further reduce endogenous BR pools in *dwf7-1* mutants. Loss of function mutations in *DWF4* result in a more severe phenotype which could be the reason why no further reduction in root length was observed in *dwf4-1* upon Pcz treatment. Alternatively, since DWF4 could be the target of Pcz – like for Brz [35] – an additional inhibition of growth can not be expected in an already genetically disrupted *dwf4-1* mutant. From our findings we conclude that BR biosynthesis mutants show a reduced sensitivity towards Pcz.

Another line of evidence that Pcz is a specific and potent BR biosynthesis inhibitor comes from transcriptional analyses of BR and GA regulated genes in *Arabidopsis*. BR homeostasis relies on the feedback regulation of *DWF4* transcription [52]. Thus, differences in *DWF4* expression reflect even minor changes in BR biosynthesis. As expected, we found that BL treatment reduced the expression of *DWF4* and other BR-biosynthetic genes in wild type, whereas Pcz application resulted in a dose-dependent increase of *DWF4*, *CPD* and *BR6ox2* transcripts (Fig. 6). The induction of *CPD* expression relative to *DWF4* and *BR6ox2* was lower upon Pcz application, however, *CPD* is primarily post-transcriptionally regulated [51]. The Pcz dependent induction of BR biosynthetic gene expression was offset by the co-application of BL similar to BL treated controls. With co-application of GA_3_ this Pcz dependent induction was not reverted (Fig. 6).

PHYB ACTIVATION TAGGED SUPPRESSOR1 (BAS1/CYP72B1) catalyzes the conversion and inactivation of BL to 26-Hydro-BL. Similar to DWF4, BAS1 is feedback regulated by endogenous BR levels [5]. We observed that BAS1 expression was induced by BL application and repressed by Pcz treatment (Fig. 6).The co-application of Pcz with BL, but not with GA_3_, countered the effect of Pcz on *BAS1* repression.

GA_20_-oxidases catalyze the sequential conversions of GA_53_ to GA_20_, late in the GA biosynthetic pathway [61]. Similar to *DWF4*, *GA20ox*-metidated steps are flux determining [62]–[63] and their expression is under feedback regulation [64]. Similar to the observations for BR-biosynthetic genes, *GA20ox1* expression appeared negatively regulated by GA_3_ (Fig. 6). However, mRNA levels of *GA20ox1* were not increased over a broad spectrum of Pcz concentrations (Fig. 6). These findings are corroborated by data from the Genevestigator database [65], which also shows a regulation of GA20-oxidases by both GA and Pac, but not Pcz. The expression pattern of GA catabolic gene *GA2ox1* [61] showed an expected induction upon GA_3_ application, but did not display relevant differences with either Pcz or BL treatments (Fig. 6). Interestingly, the GA-dependent increase in *GA2ox1* expression was impaired by simultaneous treatment with Pcz (Fig. 6). Interpreting this result, we cannot exclude the possibility of a BR-dependent GA regulation of *GA2ox1* expression. Our data also showed an overlap in the expression patterns of *BAS1* and *GA20ox1* (Fig. 6). Taken together these results do not provide evidence for a negative effect of Pcz on GA biosynthesis.

To investigate if Pcz responses found in *Arabidopsis* can be corroborated with monocot plants we chose maize, a member of the prominent grass family (*Poaceae*). This family, of close to 10,000 species, encompasses important genetic models like *Brachypodium distachyon* [66], as well as important food crops such as wheat, rice, and maize. Recently, we have shown that Pcz treatment of wild type maize phenocopies the BR deficient dwarf *nana plant1* (*na1*) and also that *na1* plants are more Pcz resistant than wild type or GA impaired mutants [23]. Using comparative treatments of increasing Pcz or Ucz concentrations we found a strong decrease in the mesocotyl length of dark-grown W22 seedlings (Fig. 7). Similar responses were detected for true leaves (Fig. 7). In comparison, the response of coleoptiles towards inhibitor treatments was less pronounced (Fig. 7). This indicates either a tissue-specific sensitivity towards Pcz or different BR levels in coleoptiles. The coleoptile, whose main role is support of juvenile leaves during soil penetration, originates directly from the pro-embryo and not from the apical meristem like the true leaves. It is therefore possible that these tissues have different reception and signaling systems for BRs.

In contrast to the results obtained with *Arabidopsis* we discovered that W22 roots were resistant towards Pcz. While Ucz treated dark-grown roots of W22 showed drastically reduced elongation, no significant response was observed over a broad range of Pcz concentrations up to 30 µM (Fig. 7). While light-grown maize seedlings are obligate heterotrophic until day 7, an equal balance between heterotrophic and autotrophically produced carbon is reached on day 10 for leaves, and day 13 to 14 for roots [67]. The slight reduction (30%) in root length observed in light-grown seedlings may be explained by the fact that these plants were measured after the switch to autotrophy when most of their carbon comes from photosynthesis. The strong reduction in plant height and decrease in photosynthetically active leaf surface at 5 µM Pcz treatment suggests that the plants had a decreased capacity to produce photosynthates. On the other hand, we analyzed dark-grown plants during a phase when they received most nutrients from the endosperm. These results may allude to fundamental differences in the control of cell elongation between W22 and *Arabidopsis* roots.

Maize roots contain the enzymes for the late C-6 oxidation steps of BR biosynthesis [68]. Our observation of differential Pcz resistance of W22 roots raised the question if this is a feature specific to W22 inbreds. We therefore tested the effect of genetic diversity in maize inbreds [54] towards Pcz response using the lines Mo20W, A619, and B73. Significant differences between these inbred lines in the length of four analyzed tissues were observed even under mock conditions. Furthermore, we found significant differences in tissue specific sensitivity towards Pcz and Ucz (Fig. 8). In general, Mo20W showed the highest sensitivity and A619 the highest resistance towards both inhibitors. Concerning tissue-specific responses, the coleoptile was the only organ which showed an even response to both Pcz and Ucz treatment in Mo20W, A619, and B73. In contrast, Pcz sensitivity in the roots and true leaves ranged from resistant (A619) to highly susceptible (B73 and Mo20W). The degree of Pcz response in maize roots seems therefore dependent on the genetic background of the maize line. The data also indicates differential hormonal regulation of tissue growth in aerial organs of maize inbreds. In rice and wheat tissue culture, accumulation of Pcz against a concentration gradient has been reported [69]. This indicates active uptake systems in these grass species. In *Monilinia fructicola*, the ABC transporter MfABC1 is induced upon Pcz treatment, which suggests a possible role for transporters of the ABC family [70] in Pcz uptake in plants and fungi [71]. Differences in either root uptake, *in planta* transport, and/or Pcz catabolism may be responsible for the observed variances between maize inbreds.

Nonetheless, our results also indicated a relation of Pcz- and BL-sensitivity between the inbred lines. Compared to Mo20W and B73, W22 and A619 plants exhibited a smaller inhibition of root elongation in the presence of either Pcz or higher concentrations of BL (Fig. 8, Fig. 9). We therefore conclude that the genetic diversity between these maize lines influences their response to BRs.

### Conclusion

We presented independent lines of evidence which indicate that Pcz inhibits BR metabolism and induces BR deficiencies in both *Arabidopsis* and maize seedlings. *Arabidopsis* seedlings treated with Pcz phenocopied BR deficient mutants such as *dwf7* and *dwf4*. Similarly, Pcz-induced dwarf phenotypes were discovered in both light and dark grown maize seedlings. Growth responses towards Pcz and Ucz were not equally expressed in all measured tissues of maize. Tissue specific sensitivity of Pcz in the coleoptile, mesocotyl, true leaves, and primary roots alludes to differential BR biosynthesis and/or signal transduction for the different maize tissues. Genetic variation of maize inbred lines implies that genetic enhancers and suppressors play a key role in Pcz-induced physiological responses. We presented that Pcz is a potent alternative to the commonly used Brz with a comparable specificity and efficacy. In contrast to Brz/Brz2001, Pcz is easily accessible and the associated costs are much lower, allowing its use for large-scale chemical genomics and field testing.

## Materials and Methods

### Plant material and growth conditions

Seeds of *Arabidopsis thaliana* were surfaced-sterilized before being sprinkled on 0.8% agar-solidified media containing 0.5× Murashige and Skoog salts and 1% sucrose. After one day of stratification at 4°C, plates were transferred to a growth room and grown under a 16 h photoperiod. For the *bzr1-1D* experiments, seedlings were stratified for 48 h at 4°C, irradiated for 6 hours to promote germination and then transferred to a growth chamber and grown in the dark at 22°C.

Maize plants were grown under greenhouse conditions at 27°C (day) and 21°C (night). Unless indicated otherwise, plants were grown in coarse Vermiculite (SunGro Horticulture, Bellevue, WA and Perlite Vermiculite Packaging Industries, Inc., North Bloomfield, OH). Plants were fertilized with 200 ppm Miracle-Gro Excel (Scotts, Marysville, OH) adjusted to pH 6 following manufacturer recommendations.

### Chemical treatments and morphometric analysis

Seedlings of 3-day old Ws-2 wild type were transferred to agar-solidified media supplemented with Pcz (Banner Maxx, Syngenta, Greensboro, NC), Brz (gift from Shozo Fujioka, Riken, Japan) and Ucz (Consise, Fine Americas Inc., Walnut Creek, CA) alone or in combination with BL (Sigma Aldrich, St Louis, MO) or GA_3_ (Gold Biotechnology, St. Louis, MO). Media plates were placed vertically to ease morphometric analysis of the root and each plate contained more than 10 seedlings. After 3 days of treatment, images were taken and the growth parameters were analyzed using ImageJ software [72]. For treatments with Pcz and Brz of BR mutants (*dwf4-1*, *dwf7-1* and *bri1-5*) and its Ws-2 wild type, the seedlings were grown for 7 days on MS media before being transferred to the inhibitor-containing media. Measurements were done after 3 days of the treatment. *bzr1-1D* and its wild type Col-0 seedlings were grown in the dark on MS media containing Pcz or Brz for 7 days and hypocotyl lengths were measured.

For all treatment experiments in maize, seeds were sterilized for 7 min at 60°C in a water bath prior to planting and grown under greenhouse conditions. For de-etiolation assays, maize seeds were imbibed for 28 h in paper towels and soaked with distilled water containing indicated concentrations of Pcz or Ucz. They were then planted 10 cm deep in 15 cm wide pots with coarse Vermiculite, watered with identical concentrations of Pcz or Ucz, and grown for additional 7 d at 28°C and 90% humidity in the dark. Control plants were grown in the dark or light in the absence of Pcz or Ucz treatment. Plants were then harvested, photographed, and analyzed using ImageJ software [72]. Mesocotyl length was determined from the root-shoot transition zone to the first node. Coleoptile and true leaf length was measured from the first node to the tip of the coleoptile or true leaves, respectively, whereas the length of the main root was used to determine root length.

For light grown Pcz experiments, W22 seeds were planted 5 cm deep in 24.5 cm wide pots with coarse Vermiculite and watered every fifth day. Pcz was added at indicated concentrations to the water solution. After 21 days plants were harvested, photographed, and analyzed using ImageJ [72]. Plant height was measured from the root-shoot transition zone to the highest leaf collar, whereas the length of the main root was used to determine root length.

### RNA extraction and qRT-PCR detection of gene transcripts

*Arabidopsis thaliana* Ws-2 seeds were surface sterilized and stratified for 48 h at 4°C, followed by growth for 4 days at 100 µmol/m^2^/sec, 16∶8 h light/dark cycle at 25°C. The seedlings were then transferred into Erlenmeyer flasks prefilled with 50 ml ½ MS with 1% sucrose liquid media (pH 5.7) and were allowed to grow for 2 d at 100 rpm under the conditions described above. For the treatments, 500× stock solutions were made in 50% DMSO (0.1% final concentration) and 100 µl of each stock solution, or 50% DMSO for the mock treatment, were applied at the beginning of the light cycle on day 7. After 10 h at 100 rpm the seedlings were harvested and immediately frozen in liquid nitrogen.

Total RNA was isolated from seedlings, as described by Eggermont et al. [73]. For qRT-PCR analysis, total RNA was pre-treated with DNase I (Invitrogen), and cDNA was synthesized using Reverse Transcriptase (Invitrogen). Ubiquitin conjugating enzyme 21 used as internal control was amplified with UBC21_FOR (300 nM) and UBC21_REV (300 nM). Gen-specific primers used were: DWF4_FOR1 (500 nM); DWF4_REV1 (500 nM); BR6ox2_FOR1 (300 nM) and BR6ox2_REV1 (300 nM); CPD_FOR1 (500 nM) and CPD_REV1 (500 nM); BAS1_FOR1 (1100 nM) and BAS1_REV1 (1100 nM); BZR1_FOR1 (300 nM) and BZR1_REV1 (300 nM); GA2ox1_FOR2 (500 nM) and GA2ox1_REV2 (500 nM) as well as GA20ox1_FOR1 (500 nM) and GA20ox1_REV1 (500 nM). Primer sequences are listed in Table S4. All primers showed >90% efficiency at their indicated concentrations. qRT-PCRs were performed as described previously [23], [74] using the StepOnePlus instrument (Invitrogen). Each data point represents the average of three independent biological replicates (approximately 30 samples per replicate), with three technical replicates.

### Statistical analysis

The Microsoft Excel^©^ add-in XL Toolbox (ver. 3.02, http://xltoolbox.sourceforge.net) was used to obtain all descriptive and comparative statistics. Analyses of variance (ANOVA) for sets of data groups were performed with “Multiple comparisons/Post-hoc” testing. Once a significant difference (*p*<0.05) was detected, “Post-hoc” tests, using the Holm-Sidak algorithm, were performed to test which of the possible multiple comparisons between the data groups were significant [75].

## Supporting Information

Table S1 **Statistical analysis of** **Figure 7 C–F** **.** Statistic analysis was performed using ANOVA with “Post Hoc” test using the Holm-Sidak algorithm. Adjusted α and adjusted *p*-values are shown and significance of *p*-values was indicated with bold text.
(DOC)

Table S2 **Statistical analysis of** **Figure 8 D–G** **.** Statistic analysis was performed using ANOVA with “Post Hoc” test using the Holm-Sidak algorithm. Adjusted α and adjusted *p*-values are shown and significance of *p*-values was indicated with bold text.
(DOC)

Table S3 **Statistical analysis of** **Figure 9** **.** Statistic analysis was performed using ANOVA with “Post Hoc” test using the Holm-Sidak algorithm. Adjusted α and adjusted *p*-values are shown and significance of *p*-values was indicated with bold text.
(DOC)

Table S4 **Oligo sequences.** Primer sequences used for qRT-PCR as described in Material and Methods. Sequences are listed from 5′ to 3′.
(DOC)

Table S5 **Number of visible leaves and leaf collars of Pcz treated W22.** W22 maize seedlings grown in the light for 3 weeks in the presence of 0, 0.2, 1, or 5 µM Pcz. All visible, including immature leaves of treated plants (*n*>13) were counted. The leaf collar was recorded if a ligule was developed. Student's *t*-test was used to obtain the indicated *p*-values for the comparison with mock.
(DOC)
