# Supplementary material for: Propiconazole Is a Specific and Accessible Brassinosteroid (BR) Biosynthesis Inhibitor for Arabidopsis and Maize
Source: PLoS One. 2012 May 9;7(5):e36625. doi: 10.1371/journal.pone.0036625 (PMC3348881; doi:10.1371/journal.pone.0036625)
Supplement: Table S1 — Statistical analysis of Figure 7 C–F . Statistic analysis was performed using ANOVA with “Post Hoc” test using the Holm-Sidak algorithm. Adjusted α and adjusted p-values are shown and significance of p-values was indicated with bold text. (DOC) [file pone.0036625.s001.doc]

Table S1

| **Compare: Treatment A** | **With: Treatment B** | **Adjusted**  **α** | **Adjusted p-Value** | **Adjusted**  **α** | **Adjusted p-Value** | **Adjusted**  **α** | **Adjusted p-Value** | **Adjusted**  **α** | **Adjusted p-Value** |
| --- | --- | --- | --- | --- | --- | --- | --- | --- | --- |
|  |  | **Mesocotyl** | **Mesocotyl** | **True Leaves** | **True Leaves** | **Coleoptile** | **Coleoptile** | **Primary Roots** | **Primary Roots** |
| Mock | Pcz 0.5 µM | 0.001507 | 0.007197 | 0.001653 | 0.249422 | 0.000789 | **0.000314** | 0.00205 | 0.067876 |
| Mock | Pcz 1 µM | 0.001192 | 0.00275 | 0.001602 | 0.241968 | 0.001114 | 0.011822 | 0.002329 | 0.128572 |
| Mock | Pcz 5 µM | 0.000814 | **1.71E-06** | 0.000986 | 0.019048 | 0.000693 | **1.6E-07** | 0.004652 | 0.482814 |
| Mock | Pcz 10 µM | 0.000765 | **1.18E-07** | 0.000789 | 0.001787 | 0.000712 | **3.97E-06** | 0.002561 | 0.145632 |
| Mock | Pcz 20 µM | 0.000702 | **1.58E-11** | 0.000657 | **2.34E-06** | 0.000657 | **2.65E-10** | 0.016952 | 0.750436 |
| Mock | Pcz 30 µM | 0.000684 | **9.59E-12** | 0.000722 | **0.00016** | 0.000666 | **5.3E-10** | 0.05 | 0.908865 |
| Mock | Ucz 0.5 µM | 0.001165 | 0.001499 | 0.001424 | 0.197723 | 0.003938 | 0.535115 | 0.005683 | 0.509958 |
| Mock | Ucz 1 µM | 0.001025 | **0.000133** | 0.000814 | 0.004127 | 0.001192 | 0.022894 | 0.00244 | 0.136834 |
| Mock | Ucz 5 µM | 0.000722 | **1.13E-08** | 0.000855 | 0.006666 | 0.001507 | 0.060886 | 0.00125 | **3.05E-05** |
| Mock | Ucz 10 µM | 6.43E-16 | **8.78E-14** | 0.000754 | **0.00059** | 0.001602 | 0.06583 | 0.000986 | **1.22E-10** |
| Mock | Ucz 20 µM | 0.000666 | **6.43E-16** | 0.000684 | **2.1E-05** | 0.000827 | **0.000663** | 0.000855 | **4.25E-13** |
| Mock | Ucz 30 µM | 0.000657 | **1.5E-17** | 0.000666 | **1.17E-05** | 0.000841 | 0.000874 | 0.000777 | **3.93E-14** |
| Pcz 0.5 µM | Pcz 1 µM | | 0.05 |  | | --- | --- | | 0.904936 | 0.007301 | 0.840523 | 0.00244 | 0.284931 | 0.008512 | 0.582348 |
| Pcz 0.5 µM | Pcz 5 µM | 0.002228 | 0.074306 | 0.001898 | 0.327268 | 0.001971 | 0.177069 | 0.003657 | 0.315326 |
| Pcz 0.5 µM | Pcz 10 µM | 0.001708 | 0.011742 | 0.001165 | 0.06142 | 0.001424 | 0.053731 | 0.005116 | 0.508592 |
| Pcz 0.5 µM | Pcz 20 µM | 0.001046 | **0.000138** | 0.000777 | 0.001192 | 0.000855 | 0.000891 | 0.002135 | 0.081065 |
| Pcz 0.5 µM | Pcz 30 µM | 0.001005 | **0.000123** | 0.001005 | 0.024657 | 0.001091 | 0.010365 | 0.001653 | 0.013992 |
| Pcz 0.5 µM | Ucz 0.5 µM | 0.016952 | 0.827927 | 0.05 | 0.937073 | 0.000869 | 0.000989 | 0.001464 | 0.004463 |
| Pcz 0.5 µM | Ucz 1 µM | 0.004265 | 0.286972 | 0.001282 | 0.095427 | 0.007301 | 0.660982 | 0.001349 | **0.001212** |
| Pcz 0.5 µM | Ucz 5 µM | 0.001349 | 0.005561 | 0.001349 | 0.144367 | 0.002135 | 0.183831 | 0.001091 | **6.69E-08** |
| Pcz 0.5 µM | Ucz 10 µM | 0.000884 | **1.31E-05** | 0.001114 | 0.059739 | 0.002846 | 0.326208 | 0.000884 | **5.94E-13** |
| Pcz 0.5 µM | Ucz 20 µM | 0.000789 | **9.7E-07** | 0.000869 | 0.00791 | 0.004652 | 0.590818 | 0.000743 | **7.97E-16** |
| Pcz 0.5 µM | Ucz 30 µM | 0.000754 | **1.07E-07** | 0.000827 | 0.00511 | 0.05 | 0.982226 | 0.000702 | **9.74E-17** |
| Pcz 1 µM | Pcz 5 µM | 0.002135 | 0.071881 | 0.001385 | 0.162245 | 0.001139 | 0.012775 | 0.006391 | 0.511639 |
| Pcz 1 µM | Pcz 10 µM | 0.001602 | 0.009281 | 0.000916 | 0.012158 | 0.001005 | 0.006781 | 0.025321 | 0.900025 |
| Pcz 1 µM | Pcz 20 µM | 0.000967 | **7.26E-05** | 0.000675 | **1.59E-05** | 0.000743 | **2.92E-05** | 0.002696 | 0.171453 |
| Pcz 1 µM | Pcz 30 µM | 0.000949 | **6.88E-05** | 0.000765 | 0.00102 | 0.000801 | **0.000375** | 0.001708 | 0.036679 |
| Pcz 1 µM | Ucz 0.5 µM | 0.010206 | 0.707162 | 0.010206 | 0.859572 | 0.001282 | 0.034609 | 0.001553 | 0.009614 |
| Pcz 1 µM | Ucz 1 µM | 0.004652 | 0.299501 | 0.000841 | 0.006184 | 0.010206 | 0.712879 | 0.001385 | 0.001773 |
| Pcz 1 µM | Ucz 5 µM | 0.001282 | 0.004315 | 0.001025 | 0.024914 | 0.008512 | 0.701544 | 0.001046 | **3.79E-08** |
| Pcz 1 µM | Ucz 10 µM | 0.000869 | **6.61E-06** | 0.000884 | 0.008514 | 0.016952 | 0.892325 | 0.000827 | **1.85E-13** |
| Pcz 1 µM | Ucz 20 µM | 0.000777 | **4.21E-07** | 0.000702 | **8.07E-05** | 0.003414 | 0.49782 | 0.000722 | **1.67E-16** |
| Pcz 1 µM | Ucz 30 µM | 0.000743 | **4.16E-08** | 0.000712 | **8.73E-05** | 0.0026960 | 0.293694 | 0.000675 | **1.88E-17** |
| Pcz 5 µM | Pcz 10 µM | 0.003414 | 0.211542 | 0.001708 | 0.252579 | 0.001898 | 0.168402 | 0.007301 | 0.559696 |
| Pcz 5 µM | Pcz 20 µM | 0.001553 | 0.009081 | 0.000949 | 0.015239 | 0.000932 | 0.003699 | 0.012741 | 0.60943 |
| Pcz 5 µM | Pcz 30 µM | 0.001464 | 0.006216 | 0.001553 | 0.236361 | 0.001653 | 0.113857 | 0.003938 | 0.337983 |
| Pcz 5 µM | Ucz 0.5 µM | 0.00183 | 0.017756 | 0.001507 | 0.207611 | 0.000702 | **6.7E-07** | 0.002846 | 0.192536 |
| Pcz 5 µM | Ucz 1 µM | 0.008512 | 0.578298 | 0.002135 | 0.405301 | 0.001708 | 0.138167 | 0.001898 | 0.056645 |
| Pcz 5 µM | Ucz 5 µM | 0.00244 | 0.109978 | 0.003013 | 0.557721 | 0.001046 | 0.007944 | 0.001221 | **2.29E-05** |
| Pcz 5 µM | Ucz 10 µM | 0.001114 | **0.000753** | 0.002228 | 0.413147 | 0.00125 | 0.031861 | 0.000967 | **1.2E-10** |
| Pcz 5 µM | Ucz 20 µM | 0.000932 | **5.95E-05** | 0.001314 | 0.126938 | 0.001314 | 0.041094 | 0.000899 | **7.77E-13** |
| Pcz 5 µM | Ucz 30 µM | 0.000841 | **3.39E-06** | 0.001221 | 0.076996 | 0.002329 | 0.248852 | 0.000789 | **7.34E-14** |
| Pcz 10 µM | Pcz 20 µM | 0.003938 | 0.241674 | 0.001971 | 0.352005 | 0.003013 | 0.360716 | 0.003013 | 0.196266 |
| Pcz 10 µM | Pcz 30 µM | 0.003201 | 0.172757 | 0.004265 | 0.704608 | 0.005683 | 0.634541 | 0.001767 | 0.046661 |
| Pcz 10 µM | Ucz 0.5 µM | 0.001139 | **0.0009** | 0.000967 | 0.017238 | 0.000754 | **3.9E-05** | 0.001602 | 0.013575 |
| Pcz 10 µM | Ucz 1 µM | 0.002561 | 0.114845 | 0.004652 | 0.709985 | 0.001464 | 0.057974 | 0.001424 | 0.002332 |
| Pcz 10 µM | Ucz 5 µM | 0.012741 | 0.744282 | 0.002846 | 0.542257 | 0.000967 | 0.005739 | 0.001068 | **4.51E-08** |
| Pcz 10 µM | Ucz 10 µM | 0.001971 | 0.040137 | 0.002696 | 0.513038 | 0.001068 | 0.008599 | 0.000814 | **1.28E-13** |
| Pcz 10 µM | Ucz 20 µM | 0.001385 | 0.005642 | 0.008512 | 0.846258 | 0.001025 | 0.007279 | 0.000712 | **1.41E-16** |
| Pcz 10 µM | Ucz 30 µM | 0.001068 | **0.000269** | 0.016952 | 0.904577 | 0.001349 | 0.045056 | 0.000666 | **1.42E-17** |
| Pcz 20 µM | Pcz 30 µM | 0.025321 | 0.900258 | 0.001139 | 0.061016 | 0.001385 | 0.053144 | 0.010206 | 0.604262 |
| Pcz 20 µM | Ucz 0.5 µM | 0.000855 | **3.83E-06** | 0.000693 | **3.18E-05** | 0.000684 | **7.37E-09** | 0.003414 | 0.28926 |
| Pcz 20 µM | Ucz 1 µM | 0.001424 | 0.005782 | 0.00125 | 0.095047 | 0.000899 | 0.001851 | 0.00183 | 0.055874 |
| Pcz 20 µM | Ucz 5 µM | 0.005683 | 0.363787 | 0.001091 | 0.058186 | 0.000732 | **2.83E-05** | 0.001139 | **1.81E-06** |
| Pcz 20 µM | Ucz 10 µM | 0.006391 | 0.446058 | 0.001068 | 0.037926 | 0.000765 | **5.93E-05** | 0.000869 | **5.89E-13** |
| Pcz 20 µM | Ucz 20 µM | 0.003013 | 0.132075 | 0.001192 | 0.070272 | 0.000722 | **1.91E-05** | 0.000754 | **1.1E-15** |
| Pcz 20 µM | Ucz 30 µM | 0.001767 | 0.012464 | 0.001464 | 0.205552 | 0.000814 | **0.000624** | 0.000693 | **6.91E-17** |
| Pcz 30 µM | Ucz 0.5 µM | 0.000801 | **1.29E-06** | 0.000801 | 0.001908 | 0.000675 | **2.36E-09** | 0.004265 | 0.457921 |
| Pcz 30 µM | Ucz 1 µM | 0.00125 | 0.003429 | 0.012741 | 0.901526 | 0.001165 | 0.017803 | 0.001971 | 0.063488 |
| Pcz 30 µM | Ucz 5 µM | 0.003657 | 0.239199 | 0.003414 | 0.622742 | 0.000777 | **0.000298** | 0.001114 | **3.07E-07** |
| Pcz 30 µM | Ucz 10 µM | 0.007301 | 0.477506 | 0.003938 | 0.664078 | 0.000916 | 0.002196 | 0.000801 | **8.26E-14** |
| Pcz 30 µM | Ucz 20 µM | 0.002846 | 0.117775 | 0.005116 | 0.718481 | 0.000884 | 0.001046 | 0.000684 | **5.09E-17** |
| Pcz 30 µM | Ucz 30 µM | 0.001314 | 0.005481 | 0.002561 | 0.436478 | 0.001221 | 0.025173 | 0.000657 | **3.46E-18** |
| Ucz 0.5 µM | Ucz 1 µM | 0.002696 | 0.116412 | 0.000899 | 0.010069 | 0.001553 | 0.061122 | 0.003201 | 0.205005 |
| Ucz 0.5 µM | Ucz 5 µM | 0.000986 | **0.000115** | 0.001046 | 0.036567 | 0.001767 | 0.138968 | 0.001165 | **2.76E-06** |
| Ucz 0.5 µM | Ucz 10 µM | 0.000732 | **2.7E-08** | 0.000932 | 0.01376 | 0.00183 | 0.1663 | 0.000932 | **1.04E-11** |
| Ucz 0.5 µM | Ucz 20 µM | 0.000712 | **5.17E-10** | 0.000732 | **0.000181** | 0.000949 | 0.004071 | 0.000765 | **4.46E-15** |
| Ucz 0.5 µM | Ucz 30 µM | 0.000693 | **1.06E-11** | 0.000743 | **0.000181** | 0.000986 | 0.005866 | 0.000732 | **4.94E-16** |
| Ucz 1 µM | Ucz 5 µM | 0.00205 | 0.051404 | 0.005683 | 0.744242 | 0.003657 | 0.531309 | 0.001314 | **0.000301** |
| Ucz 1 µM | Ucz 10 µM | 0.001091 | **0.000394** | 0.006391 | 0.804825 | 0.006391 | 0.656572 | 0.001025 | **4.23E-09** |
| Ucz 1 µM | Ucz 20 µM | 0.000916 | **3.36E-05** | 0.003657 | 0.633753 | 0.025321 | 0.921191 | 0.000916 | **3.15E-12** |
| Ucz 1 µM | Ucz 30 µM | 0.000827 | **2.22E-06** | 0.00244 | 0.430192 | 0.005116 | 0.631508 | 0.000841 | **4.09E-13** |
| Ucz 5 µM | Ucz 10 µM | 0.001898 | 0.039762 | 0.025321 | 0.925645 | 0.012741 | 0.840176 | 0.001192 | **4.97E-06** |
| Ucz 5 µM | Ucz 20 µM | 0.001221 | 0.003075 | 0.00205 | 0.396986 | 0.002561 | 0.291463 | 0.001005 | **2.97E-10** |
| Ucz 5 µM | Ucz 30 µM | 0.000899 | **2.06E-05** | 0.00183 | 0.26646 | 0.00205 | 0.180511 | 0.000949 | **2.23E-11** |
| Ucz 10 µM | Ucz 20 µM | 0.005116 | 0.331301 | 0.002329 | 0.429261 | 0.003201 | 0.440453 | 0.001507 | 0.005687 |
| Ucz 10 µM | Ucz 30 µM | 0.001653 | 0.011681 | 0.001767 | 0.255525 | 0.002228 | 0.230676 | 0.001282 | **0.000129** |
| Ucz 20 µM | Ucz 30 µM | 0.002329 | 0.083961 | 0.003201 | 0.602766 | 0.004265 | 0.560554 | 0.002228 | 0.096526 |
