# Supplementary material for: Propiconazole Is a Specific and Accessible Brassinosteroid (BR) Biosynthesis Inhibitor for Arabidopsis and Maize
Source: PLoS One. 2012 May 9;7(5):e36625. doi: 10.1371/journal.pone.0036625 (PMC3348881; doi:10.1371/journal.pone.0036625)
Supplement: Table S2 — Statistical analysis of Figure 8 D–G . Statistic analysis was performed using ANOVA with “Post Hoc” test using the Holm-Sidak algorithm. Adjusted α and adjusted p-values are shown and significance of p-values was indicated with bold text. (DOC) [file pone.0036625.s002.doc]

|  |  | **Mesocotyl** | | | |  | **True Leaves** | | |
| --- | --- | --- | --- | --- | --- | --- | --- | --- | --- |
| **Maize Inbred** | **Compare: Treatment A** | | **With: Treatment B** | **Adjusted**  **α** | **Adjusted**  **p-Value** | **Compare: Treatment A** | **With: Treatment B** | **Adjusted**  **α** | **Adjusted**  **p-Value** |
| B73 | Mock | | Pcz 1 µM | 0.000943 | **4.95E-07** | Mock | Pcz 1 µM | 0.000676 | 0.000908 |
| B73 | Mock | | Pcz 10 µM | 0.000667 | **5.24E-11** | Mock | Pcz 10 µM | 0.000543 | **4.75E-06** |
| B73 | Mock | | Ucz 1 µM | 0.000633 | **5.79E-12** | Mock | Ucz 1 µM | 0.000485 | **9E-10** |
| B73 | Mock | | Ucz 10 µM | 0.000549 | **2.23E-15** | Mock | Ucz 10 µM | 0.000476 | **4.09E-12** |
| B73 | Pcz 1 µM | | Pcz 10 µM | 0.002083 | 0.002611 | Pcz 1 µM | Pcz 10 µM | 0.001136 | 0.087421 |
| B73 | Pcz 1 µM | | Ucz 1 µM | 0.002273 | 0.02198 | Pcz 1 µM | Ucz 1 µM | 0.000694 | 0.001506 |
| B73 | Pcz 1 µM | | Ucz 10 µM | 0.000877 | **6.48E-08** | Pcz 1 µM | Ucz 10 µM | 0.000562 | **1.56E-05** |
| B73 | Pcz 10 µM | | Ucz 1 µM | 0.002778 | 0.053837 | Pcz 10 µM | Ucz 1 µM | 0.001471 | 0.218863 |
| B73 | Pcz 10 µM | | Ucz 10 µM | 0.001786 | **0.000247** | Pcz 10 µM | Ucz 10 µM | 0.000794 | 0.009207 |
| B73 | Ucz 1 µM | | Ucz 10 µM | 0.000794 | **1.05E-09** | Ucz 1 µM | Ucz 10 µM | 0.000847 | 0.027365 |
| Mo20W | Mock | | Pcz 1 µM | 0.000538 | **1.06E-15** | Mock | Pcz 1 µM | 0.000581 | **3.82E-05** |
| Mo20W | Mock | | Pcz 10 µM | 0.000575 | **2.11E-14** | Mock | Pcz 10 µM | 0.000625 | **0.00025** |
| Mo20W | Mock | | Ucz 1 µM | 0.000556 | **3.64E-15** | Mock | Ucz 1 µM | 0.000575 | **2.8E-05** |
| Mo20W | Mock | | Ucz 10 µM | 0.0005 | **1.91E-21** | Mock | Ucz 10 µM | 0.000556 | **1.56E-05** |
| Mo20W | Pcz 1 µM | | Pcz 10 µM | 0.025 | 0.77696 | Pcz 1 µM | Pcz 10 µM | 0.0125 | 0.945157 |
| Mo20W | Pcz 1 µM | | Ucz 1 µM | 0.008333 | 0.69866 | Pcz 1 µM | Ucz 1 µM | 0.002083 | 0.513043 |
| Mo20W | Pcz 1 µM | | Ucz 10 µM | 0.001351 | **6.77E-05** | Pcz 1 µM | Ucz 10 µM | 0.003846 | 0.849379 |
| Mo20W | Pcz 10 µM | | Ucz 1 µM | 0.007143 | 0.54607 | Pcz 10 µM | Ucz 1 µM | 0.002 | 0.502329 |
| Mo20W | Pcz 10 µM | | Ucz 10 µM | 0.001613 | **0.000232** | Pcz 10 µM | Ucz 10 µM | 0.00625 | 0.921501 |
| Mo20W | Ucz 1 µM | | Ucz 10 µM | 0.001429 | **0.000143** | Ucz 1 µM | Ucz 10 µM | 0.001613 | 0.343602 |
| A619 | Mock | | Pcz 1 µM | 0.004545 | 0.291626 | Mock | Pcz 1 µM | 0.001389 | 0.151689 |
| A619 | Mock | | Pcz 10 µM | 0.00098 | **8.63E-07** | Mock | Pcz 10 µM | 0.001316 | 0.131045 |
| A619 | Mock | | Ucz 1 µM | 0.000909 | **1.97E-07** | Mock | Ucz 1 µM | 0.001 | 0.047373 |
| A619 | Mock | | Ucz 10 µM | 0.000588 | **4.58E-14** | Mock | Ucz 10 µM | 0.000649 | **0.000417** |
| A619 | Pcz 1 µM | | Pcz 10 µM | 0.001111 | **7.73E-06** | Pcz 1 µM | Pcz 10 µM | 0.005 | 0.892796 |
| A619 | Pcz 1 µM | | Ucz 1 µM | 0.000962 | **5.44E-07** | Pcz 1 µM | Ucz 1 µM | 0.002778 | 0.598487 |
| A619 | Pcz 1 µM | | Ucz 10 µM | 0.000581 | **3.45E-14** | Pcz 1 µM | Ucz 10 µM | 0.000862 | 0.028424 |
| A619 | Pcz 10 µM | | Ucz 1 µM | 0.003571 | 0.130403 | Pcz 10 µM | Ucz 1 µM | 0.003333 | 0.711453 |
| A619 | Pcz 10 µM | | Ucz 10 µM | 0.000781 | **9.1E-10** | Pcz 10 µM | Ucz 10 µM | 0.000962 | 0.04351 |
| A619 | Ucz 1 µM | | Ucz 10 µM | 0.001282 | **3.4E-05** | Ucz 1 µM | Ucz 10 µM | 0.001042 | 0.058878 |

Table S2
