# Supplementary material for: Propiconazole Is a Specific and Accessible Brassinosteroid (BR) Biosynthesis Inhibitor for Arabidopsis and Maize
Source: PLoS One. 2012 May 9;7(5):e36625. doi: 10.1371/journal.pone.0036625 (PMC3348881; doi:10.1371/journal.pone.0036625)
Supplement: Table S3 — Statistical analysis of Figure 9 . Statistic analysis was performed using ANOVA with “Post Hoc” test using the Holm-Sidak algorithm. Adjusted α and adjusted p-values are shown and significance of p-values was indicated with bold text. (DOC) [file pone.0036625.s003.doc]

| **Maize**  **inbred** | **Compare:**  **Treatment**  **A** | **With:**  **Treatment**  **B** | **Adjusted**  **α** | **Adjusted**  **p-value** |
| --- | --- | --- | --- | --- |
| W22 | Mock | 1.25 µM PCZ | 0.002696 | 0.152842 |
| W22 | Mock | 5 µM PCZ | 0.000949 | **9.13E-05** |
| W22 | Mock | 20 µM PCZ | 0.000618 | **1.62E-08** |
| W22 | 1.25 µM PCZ | 5 µM PCZ | 0.001971 | 0.050435 |
| W22 | 1.25 µM PCZ | 20 µM PCZ | 0.000916 | **8.29E-05** |
| W22 | 5 µM PCZ | 20 µM PCZ | 0.001068 | **0.000907** |
| Mo20W | Mock | 1.25 µM PCZ | 0.003414 | 0.199337 |
| Mo20W | Mock | 5 µM PCZ | 0.000801 | **4.34E-06** |
| Mo20W | Mock | 20 µM PCZ | 0.000484 | **7.29E-17** |
| Mo20W | 1.25 µM PCZ | 5 µM PCZ | 0.001139 | 0.001724 |
| Mo20W | 1.25 µM PCZ | 20 µM PCZ | 0.000529 | **5.70E-12** |
| Mo20W | 5 µM PCZ | 20 µM PCZ | 0.000675 | **3.68E-07** |
| A619 | Mock | 1.25 µM PCZ | 0.001653 | 0.027632 |
| A619 | Mock | 5 µM PCZ | 0.001091 | 0.001550 |
| A619 | Mock | 20 µM PCZ | 0.000493 | **5.12E-15** |
| A619 | 1.25 µM PCZ | 5 µM PCZ | 0.003657 | 0.228995 |
| A619 | 1.25 µM PCZ | 20 µM PCZ | 0.000576 | **2.14E-10** |
| A619 | 5 µM PCZ | 20 µM PCZ | 0.000722 | **8.64E-07** |
| B73 | Mock | 1.25 µM PCZ | 0.002329 | 0.076811 |
| B73 | Mock | 5 µM PCZ | 0.000583 | **3.91E-10** |
| B73 | Mock | 20 µM PCZ | 0.000450 | **8.13E-20** |
| B73 | 1.25 µM PCZ | 5 µM PCZ | 0.000684 | **4.15E-07** |
| B73 | 1.25 µM PCZ | 20 µM PCZ | 0.000466 | **1.42E-17** |
| B73 | 5 µM PCZ | 20 µM PCZ | 0.000589 | **8.76E-10** |

Table S3
