# Supplementary material for: Propiconazole Is a Specific and Accessible Brassinosteroid (BR) Biosynthesis Inhibitor for Arabidopsis and Maize
Source: PLoS One. 2012 May 9;7(5):e36625. doi: 10.1371/journal.pone.0036625 (PMC3348881; doi:10.1371/journal.pone.0036625)
Supplement: Table S4 — Oligo sequences. Primer sequences used for qRT-PCR as described in Material and Methods. Sequences are listed from 5′ to 3′. (DOC) [file pone.0036625.s004.doc]

| **Primer** | **Sequence (5’ to 3’)** |
| --- | --- |
| CPD_FOR1 | TTACCGCAAAGCCATCCAA |
| CPD_REV1 | TCATCACCACCACCACCGTCAAC |
| DWF4_FOR1 | GTTGGCCATTTCTTGGTGAAA |
| DWF4_REV1 | TGGCGGTGTACGGTTTAAGA |
| GA20ox1_FOR1 | GAAAATCAATGGCGCGCTCCAT |
| GA20ox1_REV1 | CGGTGCAAGCAGCTCTTGTA |
| GA2ox1_FOR2 | TGTCCCTCCCGATCACACTT |
| GA2ox1_REV2 | ACGCTCTTGAACCTCCCATTT |
| BAS1_FOR1 | CATGTGGCACGGAGCTTCTA |
| BAS1_REV1 | CCGAGCTGGATTGAATTCGT |
| BZR1_FOR1 | CAAACCCAAATGGCATGTCA |
| BZR1_REV1 | CCAAATACCTGGACGAAGAAGAA |
| BR6ox2_FOR1 | AACCGCTCACTCTCGACGAT |
| BR6ox2_REV1 | CGTTGCCAATCTTGATGTCTCA |
| UBC21_FOR | CTGCGACTCAGGGAATCTTCTAA |
| UBC21_REV | TTGTGCCATTGAATTGAACCC |

Table S4
