# Supplementary material for: Propiconazole Is a Specific and Accessible Brassinosteroid (BR) Biosynthesis Inhibitor for Arabidopsis and Maize
Source: PLoS One. 2012 May 9;7(5):e36625. doi: 10.1371/journal.pone.0036625 (PMC3348881; doi:10.1371/journal.pone.0036625)
Supplement: Table S5 — Number of visible leaves and leaf collars of Pcz treated W22. W22 maize seedlings grown in the light for 3 weeks in the presence of 0, 0.2, 1, or 5 µM Pcz. All visible, including immature leaves of treated plants (n>13) were counted. The leaf collar was recorded if a ligule was developed. Student's t-test was used to obtain the indicated p-values for the comparison with mock. (DOC) [file pone.0036625.s005.doc]

| **Treatment** | **No. of leaves** | ***p* value** | **No. of leaf collars** | ***p* value** |
| --- | --- | --- | --- | --- |
| Mock | 4 ± 0.19 |  | 2.30 ± 0.46 |  |
| 0.2 µM Pcz | 4.08 ± 0.28 | 0.087 | 2 ± 0.51 | 0.026 |
| 1 µM Pcz | 3.96 ± 0.2 | 0.939 | 1.83 ± 0.38 | 0.0003 |
| 5 µM Pcz | 4 ± 0.41 | 0.433 | 1.69 ± 0.48 | 0.001 |

Table S5
